# 246. A Retrospective Cohort Study Comparing Two Versus Three Blood Culture Sets in People Who Inject Drugs

**DOI:** 10.1093/ofid/ofad500.319

**Published:** 2023-11-27

**Authors:** Aprilgate Wang, Michael C Shih, Stella Radosta, David Mushatt

**Affiliations:** Tulane University School of Medicine, New Orleans, Louisiana; Tulane University School of Medicine, New Orleans, Louisiana; Tulane University School of Medicine, New Orleans, Louisiana; Tulane University School of Medicine, New Orleans, Louisiana

## Abstract

**Background:**

The current standard of drawing two versus three sets of blood cultures lacks adequate guidance. Because people who inject drugs (PWID) are at higher risk for bacteremia and life-threatening illness, risk-benefit analysis become especially unclear.

**Figure:**
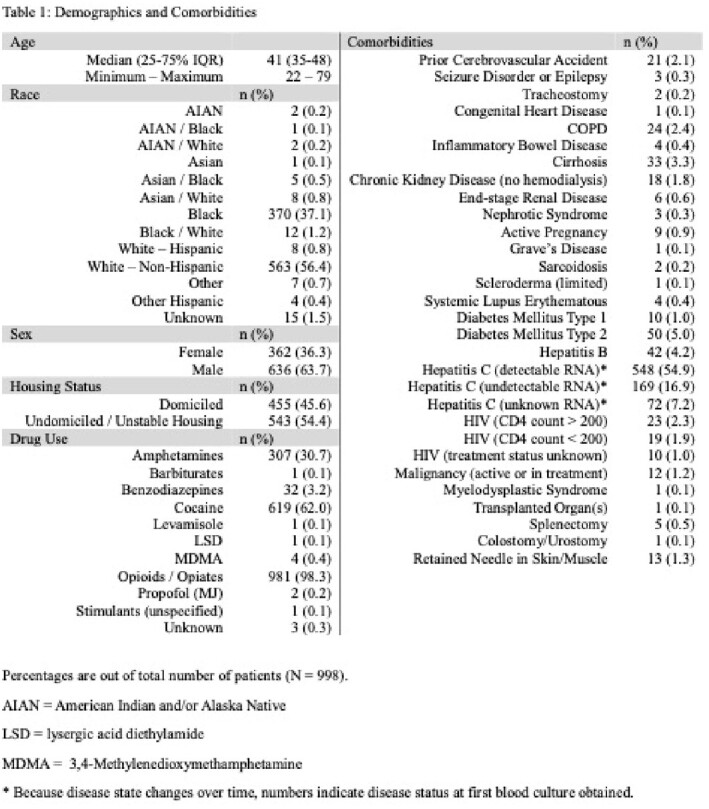

**Methods:**

We conducted a retrospective cohort study of PWID who had blood cultures drawn between 2017 and 2022 at a single multihospital system. Data was extracted and analyzed to determine (a) the rates of bacteremia for two versus three sets of blood cultures, (b) rates of false positive (contaminants) for two versus three sets of blood cultures, and (c) the downstream effects from contaminant growths. Exclusion criteria were blood cultures obtained greater than four hours apart, blood cultures obtained after antibiotic administration, and subsequent blood cultures obtained during the same hospitalization.

**Results:**

998 PWID patients with 2278 sets of blood cultures were analyzed. There were 1618 cases with two blood culture sets and 660 cases with three. Potential benefit of adding a third blood culture set, indicated by 1 out of 3 containing pathogenic growth, was seen in 30 (4.5%) cases. However, only 13 (2.0%) cases showed true benefit, as 17 (2.6%) involved known inadequately treated infections or the same pathogen on another culture. The number needed to treat (NNT) was 51. By adding a third blood culture set, the relative risk of a contaminant increased by 39.7%; the number needed to harm (NNH) was 36. There were statistically more contaminants in three blood culture sets (65, 9.8%) than for two (114, 7.1%) (*p<*0.00001). Out of 179 culture sets with only contaminants, 115 (64.2%) were analyzed for complications, which included 7 (6.14%) hospital readmissions, average 3.4 (SD 1.9) extra days of admission for 9 patients, average 1.3 (SD 1.2) extra blood cultures, total 13 (11.9%) extra microbial speciations, and average 2.3 (SD 1.9) days of unnecessary antibiotic administration for 27 patients.

**Figure:**
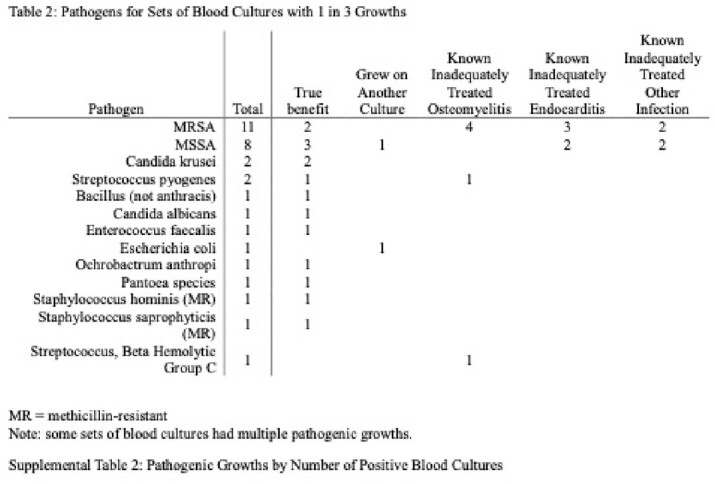

**Figure:**
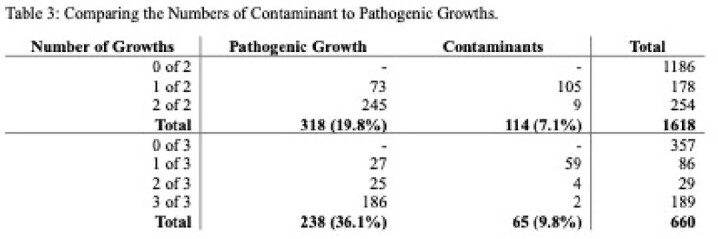

**Figure:**
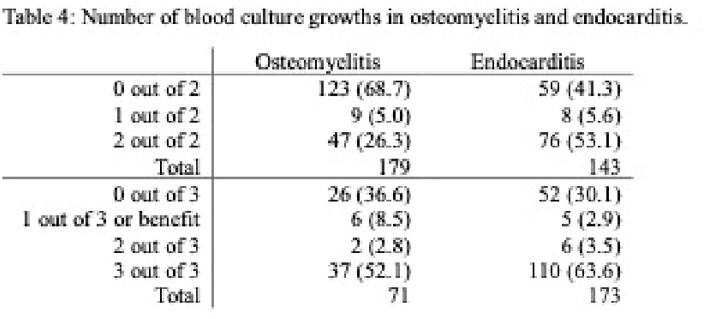

**Conclusion:**

Benefits of a third blood culture set do not universally outweigh risks for contaminant growth for PWID. A third blood culture set should be considered in specific clinical scenarios (i.e., inadequately treated endocarditis and osteomyelitis).

**Figure:**
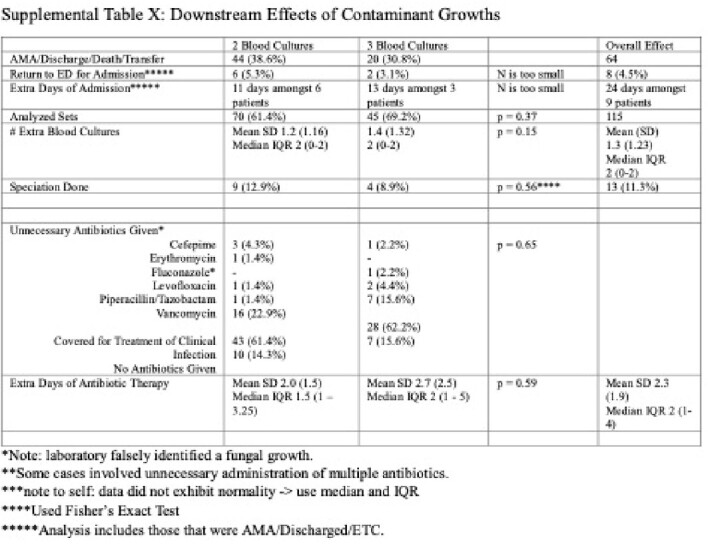

**Figure:**
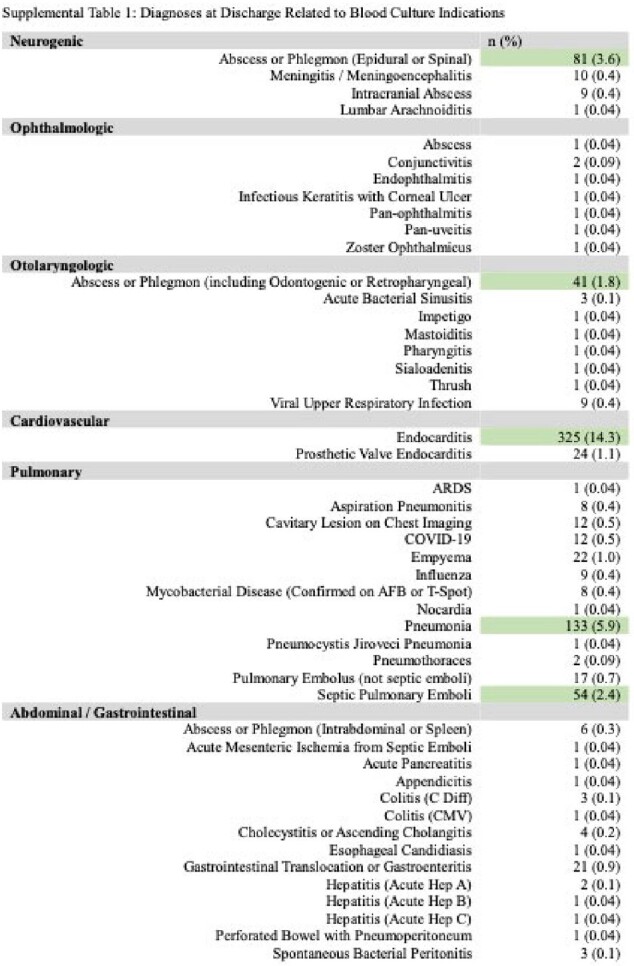

**Disclosures:**

**All Authors**: No reported disclosures

